# Untargeted metabolomics analysis of the hippocampus and cerebral cortex identified the neuroprotective mechanisms of Bushen Tiansui formula in an aβ_25-35_-induced rat model of Alzheimer’s disease

**DOI:** 10.3389/fphar.2022.990307

**Published:** 2022-10-20

**Authors:** Hongli Li, Yejun Tan, Xin Cheng, Zheyu Zhang, Jianhua Huang, Shan Hui, Lemei Zhu, Yuqing Liu, Di Zhao, Zhao Liu, Weijun Peng

**Affiliations:** ^1^ Department of Integrated Traditional Chinese and Western Medicine, The Second Xiangya Hospital, Central South University, Changsha, China; ^2^ National Clinical Research Center for Mental Disorder, Changsha, China; ^3^ School of mathematics, University of Minnesota twin Cities, St. Paul, MS, United States; ^4^ Hunan Academy of Chinese Medicine, Changsha, China; ^5^ Department of Geratology, Hunan Provincial People’s Hospital, The First Affiliated Hospital of Hunan Normal University, Changsha, China; ^6^ Academician Workstation, Changsha Medical University, Changsha, China

**Keywords:** Alzheimer’s disease, Bushen Tiansui formula, cerebral cortex, hippocampus, metabolomics

## Abstract

**Background:** Bushen Tiansui Formula (BSTSF) is a traditional formulation of Chinese medicine that has been used to treat Alzheimer’s disease (AD) for decades; however, the underlying mechanisms by which this formula achieves such therapeutic effects have yet to be elucidated.

**Prupose:** To investigate the neuroprotective mechanisms of BSTSF against AD by analyzing metabolite profiles in the hippocampus and cortex of AD rats.

**Methods:** The rat models of AD were established by the injection of Aβ_25–35_. The Morris water maze (MWM) test was performed to evaluate the effect of BSTSF treatment on cognitive dysfunction. Hematoxylin and eosin (HE) staining was used to assess the effect of BSTSF on typical AD pathologies. Underlying mechanisms were investigated using LC-MS/MS-based untargeted metabolomics analysis of the cerebral cortex and hippocampus.

**Results:** BSTSF significantly improved memory deficits and the typical histopathological changes of AD rats. Untargeted metabolomics analysis showed that 145 and 184 endogenous metabolites in the cerebral cortex and hippocampus, respectively, were significantly different in the BSTSF group when compared with the AD group. The differential metabolites in the cerebral cortex were primarily involved in cysteine and methionine metabolism, while those in the hippocampus were mainly involved in d-Glutamine and d-glutamate metabolism.

**Conclusion:** In the present study, we confirmed the neuroprotective effects of BSTSF treatment against AD using a rat model. Our findings indicate that the BSTSF-mediated protective effects were associated with amelioration of metabolic disorders in the hippocampus and cerebral cortex.

## 1 Introduction

Alzheimer’s disease (AD) is the most common form of dementia characterized by neurodegeneration and memory loss (Belfiore et al., 2019). At the present time, more than 47 million people are suffering from AD across the world; this number is expected to rise to 131 million by 2050 (Su et al., 2022). As the global society ages, AD has become a serious threat to humanity (Serneels et al., 2020). However, there is no effective treatment available for AD. Therefore, developing new therapies to treat AD is becoming ever more urgent.

Traditional Chinese Medicine (TCM) has been recognized and accepted as a potential and promising approach for the treatment of AD. Bushen Tiansui Formula (BSTSF, also known as “Naoling decoction”), a traditional formula of TCM is derived from Sagacious Confucius’ Pillow Elixir (Yi et al., 2020) and has been commonly used to treat AD in China for decades (Hou et al., 2019; Yi et al., 2020; Zhang et al., 2020). We have previously demonstrated that BSTSF can be used for the treatment of AD, potentially by ameliorating synaptic impairment by upregulating the expression levels of brain-derived neurotrophic factor (BDNF) and attenuating nerve inflammation (Hui et al., 2017; Xia et al., 2017). However, the neuroprotective mechanisms of BSTSF remain largely unclear, particularly in terms of the regulatory function of brain metabolism.

Metabolomics, as an important branch of the omics technologies, aims to provide a global understanding of metabolites in integrated living systems and dynamic responses to changes in both endogenous and exogenous factors. Numerous rapid and high-throughput techniques, such as nuclear magnetic resonance (NMR), liquid chromatography tandem mass spectrometry (LC-MS) and gas chromatography mass spectrometry (GC-MS) have been used in metabolomic studies (Cui et al., 2018). Due to its high sensitivity, versatility, and no need for chemical derivatization, and with the introduction of new ionization techniques, LC-MS/MS-based metabolomics has gained popularity in recent years (Shi et al., 2021). This technology could provide a new perspective for understanding the complicated mechanisms of diseases and for evaluating drug effects from a comprehensive and holistic point of view (Shao et al., 2020; Liu et al., 2021). Undoubtedly, metabolomics could provide integrative platforms to give a comprehensive interpretation of TCM mechanisms from a modern biological and medical view. The cerebral cortex and hippocampus are the earliest affected brain regions in AD (Lazarov and Hollands, 2016; Zheng et al., 2019). Increasing lines of evidence have demonstrated that metabolic disorders in the cerebral cortex and hippocampus play an important role in the pathogenesis of AD (Crist et al., 2021). Targeting these metabolic disorders in the cerebral cortex and hippocampus has emerged as a promising therapeutic strategy for AD. Therefore, we hypothesized that untargeted metabolomics of cerebral cortex and hippocampus is an exciting option for investigating the neuroprotective mechanisms of BSTSF.

In the study, we aimed to investigate whether BSTSF could regulate metabolic disorders of the cerebral cortex and hippocampus in AD, and to further elucidate the underlying neuroprotective mechanisms of BSTSF. First, we generated a rat model of AD by intracerebroventricularly injecting Aβ_25-35_. Next, we performed the Morris water maze (MWM) test to evaluate the effect of BSTSF on cognitive performance. Hematoxylin and eosin (HE) staining was conducted to evaluate the effect of BSTSF on histopathological changes. Finally, the role of BSTSF in regulating metabolic disorders in the cerebral cortex and hippocampus was revealed by untargeted metabolomics.

## 2 Materials and methods

### 2.1 Preparation of lyophilized BSTSF powder

The six herbal ingredients of BSTSF were purchased from Tongrentang (Beijing, China) and authenticated by Professor Sifang Zhang from the Second Xiangya Hospital of Central South University. The voucher specimens (Number TCM 20200369) were deposited at the Second Xiangya Hospital of Central South University. The six herbs *Epimedium acuminatum* Franch. (Yin-yang-huo), *Fallopia multiflora* (Thunb.) Harald. (He-shou-wu), Polygala tenuifolia Willd. (Yuan-zhi), *Acorus tatarinowii* Schott. (Shi-chang-pu), *Plastrum testudinis* (Gui-ban), and *Ossa draconis* (Long-gu), were dried and ground into a crude powder and mixed in proportion to obtain BSTSF. The freeze-dried BSTSF powder was prepared according to our previous publication (Yi et al., 2020). Briefly, the herbs were extracted under reflux with water for 2 h and 1.5 h, respectively. Then, the filtrates were pooled and dried using a vacuum-assisted freeze-drying apparatus, the obtained lyophilized powder was stored at 4°C. And the lyophilized powder of BSTSF was dissolved into a solution with distilled water for gavage. BSTSF was analyzed by LC/MS-Q-TOF in our previous study. The results showed that 2,3,5,4′-tetrahydroxystilbene-2-O-β-d-glucopyranoside (THSG) and Icariin were detected as primary components in BSTSF (Xia et al., 2017).

### 2.2 Animal modelling and treatment

Aβ_25–35_ was purchased from Sigma Chemicals (Sigma–Aldrich, Saint Louis, United States) and dissolved in deionized water at a concentration of 4 μg/μl. Adult male Sprague-Dawley (SD) rats (Hunan SJA Laboratory Animal Co. Ltd) weighing 180–220 g were used in this study. All rats were pathogen- and virus-free. After 7 days of adaptive feeding, rats were randomly divided into three groups: sham group, AD group, and BSTSF group. The AD rat model was induced as reported previously (Chen et al., 2021). In brief, rats were anesthetized and placed in a stereotaxic device. Rats in the AD group were injected with 5 μl of Aβ_25–35_ into the right lateral ventricle. Rats in the sham group were injected with sterile normal saline. Subsequently, the rats received oral gavage for 4 weeks. The BSTSF groups were orally administered with BSTSF at a dose of 27 g/kg/d; the two other groups were given the same volume of sterile normal saline. Our previous study explored the efficacy of three doses (9 g/kg/d, 27 g/kg/d, and 54 g/kg/d) of BSTSF, and the result revealed that this prescription owns optimal efficacy when it is administered at 27 g/kg/d; therefore, a dose of 27 g/kg/d was chosen for the experiments in the current study (Xia et al., 2017). All treatments were approved by the ethics committee of the Central South University (reference: 2020–0031).

### 2.3 Morris water maze task

The MWM was used to evaluate spatial learning and memory and was performed as described previously (Dinel et al., 2020). In brief, the procedure consisted of 5 days (24–28 days after Aβ_25-35_ infusion) of memory acquisition experiments and 1 day (day 29 after Aβ_25-35_ infusion) of spatial probe experiments. Behavior was automatically video-recorded by automated video tracking. The Super Maze Morris system (XR-XM101, Shanghai Softmaze Information Technology Co. Ltd., China) was used for recording and analysis.

### 2.4 Hematoxylin and eosin staining

At the end of the MWM tasks, the rats were anesthetized and perfused transcardially with 300 ml of stroke-physiological saline solution to remove blood. Brain tissue was harvested, fixed with 4% paraformaldehyde solution, embedded in paraffin, and cut into sections that were 4-5 µm thick. Then, the sections were stained with hematoxylin and eosin solution and sealed with neutral gum. Finally, pathological changes in the brain tissue were observed and photographed with an optical microscope.

### 2.5 Sample preparations for metabolomics

After the MWM tests, we harvested brain tissue and isolated the cerebral cortex and hippocampus; these were then stored at -80°C. Next, 30 mg samples were weighed and homogenized with 400 μl of methanol:water (4:1, v/v) solution. Then, the samples were vortexed, sonicated and centrifuged at 13000 rpm at 4°C for 15 min. The supernatants were then transferred to sample vials. We also prepared a quality control (QC) sample from pooled supernatants.

### 2.6 UPLC-Q-Exactive-MS/MS based metabolomics

#### 2.6.1 Acquisition of LC-MS/MS data

UPLC-MS/MS analysis was performed on a Q-Exactive LC-MS/MS system (Thermo Scientific, Bremen, Germany). Chromatographic separations were performed on a ACQUITY UPLC HSS T3 column (100 mm × 2:1 mm, 1.8 μm). The mobile phase consisted of solvent A (0.1% formic acid in water) and 0.1% solvent B acetonitrile). The solvent gradient program was as follows: 0-3min, 95% (A): 5% (B) - 70% (A): 30% (B); 3-5min, 70% (A): 30% (B)—40% (A): 60% (B); 5-7min, 40% (A): 60% (B)—20% (A): 80% (B); 7-12min, 20% (A): 80% (B) - 0% (A): 100% (B); 12-16.5min, 0% (A): 100% (B)—95% (A): 5% (B). The column temperature was 45°C. The sample injection volume was 10 μl and the flow rate was 0.35 ml/min. All samples were stored at 4°C during analysis. The source temperature was 320°C and the declustering potential was 80 V. The shealth gas flow rate was set to 30 L/min in both positive and negative ion modes. The electrospray voltage was 3.5 kV in positive-ion mode and 3.1 kV in negative-ion mode.

### 2.6.2 Data processing and analysis

Pre-processing and analysis of the UPLC-MS data were performed as described previously (Yi et al., 2020). In brief, the data were collected in both positive and negative mode. All raw data were then loaded into Progenesis QI software (Waters, Milford, MA, United States) and SIMCA-P14.0 software (Umetrics AB, Umea, Vasterbotten, Sweden) for further analysis.

In order to conduct multivariate statistical analysis, Majorbio Cloud Platform (https://cloud.majorbio.com) and SIMCA-P 14.1(Umetrics, Sweden) was carried out for the principal components analysis (PCA) and orthogonal partial least-squares discriminate (OPLS-DA) analysis. Variable importance in the projection (VIP) > 1 and *p* < 0.05 were selected as candidate metabolites. Besides, significantly altered metabolite data were imported into MetaboAnalyst 5.0 to investigate the neuroprotective mechanisms of BSTSF treatment on AD. The impact value threshold calculated from pathway topology analysis was set to 0.10, and a raw *p* value <0.05 was regarded as significant.

### 2.7 Statistical analysis

Data are reported as the mean ± standard deviation (SD) and were analyzed by SPSS 20.0 software (IBM, Armonk, NY, United States). The escape latency was analyzed by separate repeated measures two-way analysis of variance (ANOVA). Other data were evaluated by one-way ANOVA. *p* < 0.05 was considered statistically significant.

## 3 Results

### 3.1 BSTSF improves cognitive impairment in Aβ_25-35_-induced AD rats

**FIGURE 1:**
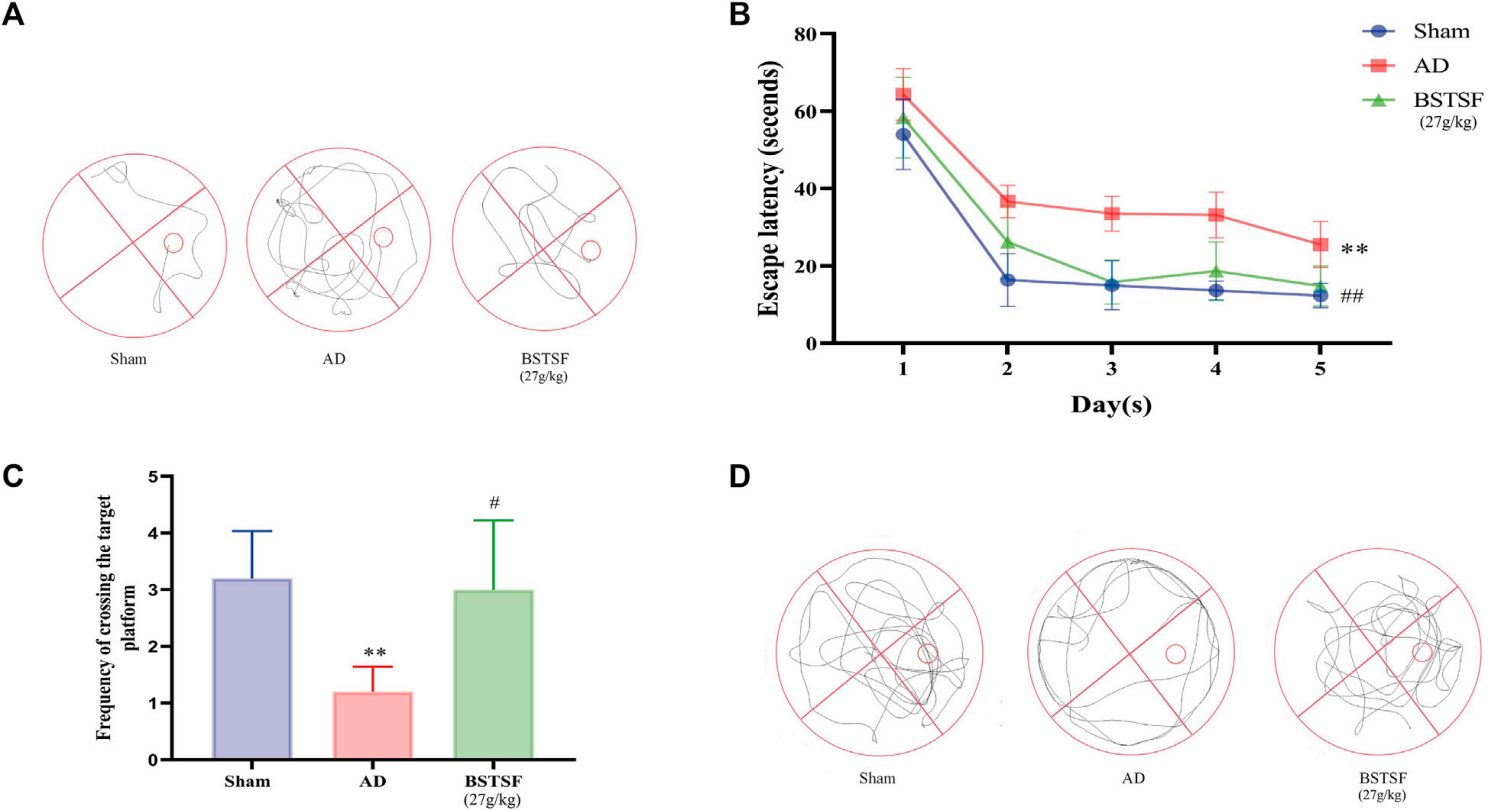
BSTSF ameliorates cognitive deficiency. **(A)** representative images of swimming paths; **(B)** time spent finding the hidden platform; **(C)** the number of platform crossings; **(D)** representative images of the frequency of motion track. Data are expressed as the mean ± SD (n = 5). ∗∗*p* < 0.01 *vs*. sham group; #*p* < 0.05, ##*p* < 0.01 *vs*. AD group.

To investigate the effect of BSTSF on learning and spatial memory in AD rats, the MWM tests were performed 28 days after administration. As shown by the MWM tests, in the AD group, the swimming trajectory was regular and single, the number of platform crossings were reduced, and the escape latency was longer than that in the sham group (*p* < 0.01). However, in the BSTSF group, the swimming trajectory was disorderly, the number of platform crossings were increased (*p* < 0.05), and the escape latency was shorter than in the AD group (*p* < 0.01) (Figure 1). These results revealed that the AD rats exhibited impaired spatial cognition and that BSTSF treatment significantly alleviated the cognitive deficits of AD rats.

### 3.2 BSTSF alleviated histopathological changes in the brains of Aβ_25-35_-induced AD rats

**FIGURE 2:**
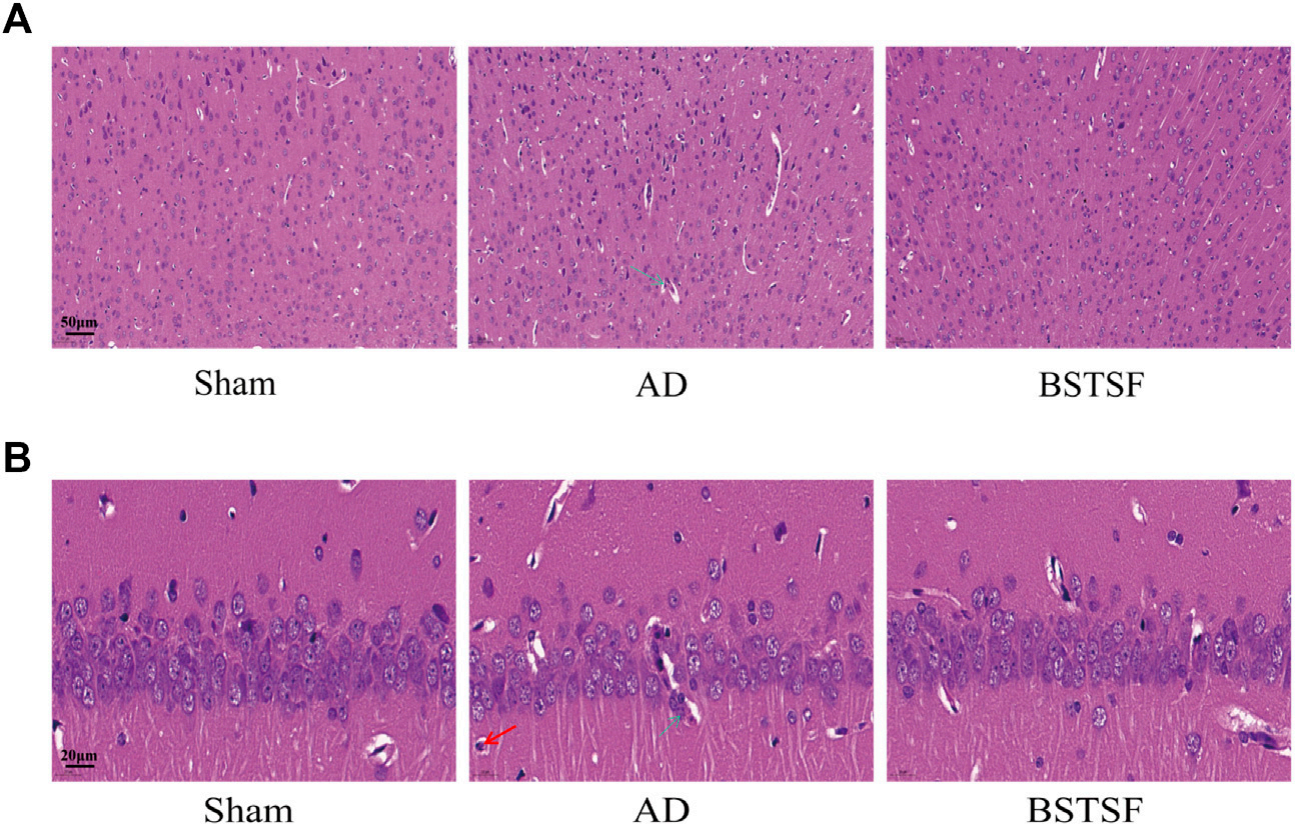
Representative images of HE staining in the cerebral cortex **(A)** and hippocampus **(B)**. The magnification: **(A)** 20×, **(B)** 63×. The green arrows indicate sites of structural disorder and hyperemia, the red arrows indicate sites of the with pale and oval nucleus and more cytoplasm.

Morphological changes in the brains of experimental rats were examined by HE staining. As shown in Figure 2, the hippocampus and cortex neurons were organically aligned in the sham group; there was no evidence of pyknosis, hyperemia or swelling. However, in the AD rats, the cell density was decreased, cell arrangement was disordered, and there was clear evidence of pyknosis, hyperemia and swelling. After 28 days of treatment, it was evident that BSTSF had significantly prevented these pathological changes; the nerve cells were organically aligned and there was no evidence of pyknosis, hyperemia or swelling in the BSTSF group.

### 3.3 BSTSF treatment improved AD-related changes in metabolomic profiles of the cerebral cortex and hippocampus in a rat model of AD

#### 3.3.1 Multivariate statistical analysis of metabolites

**FIGURE 3:**
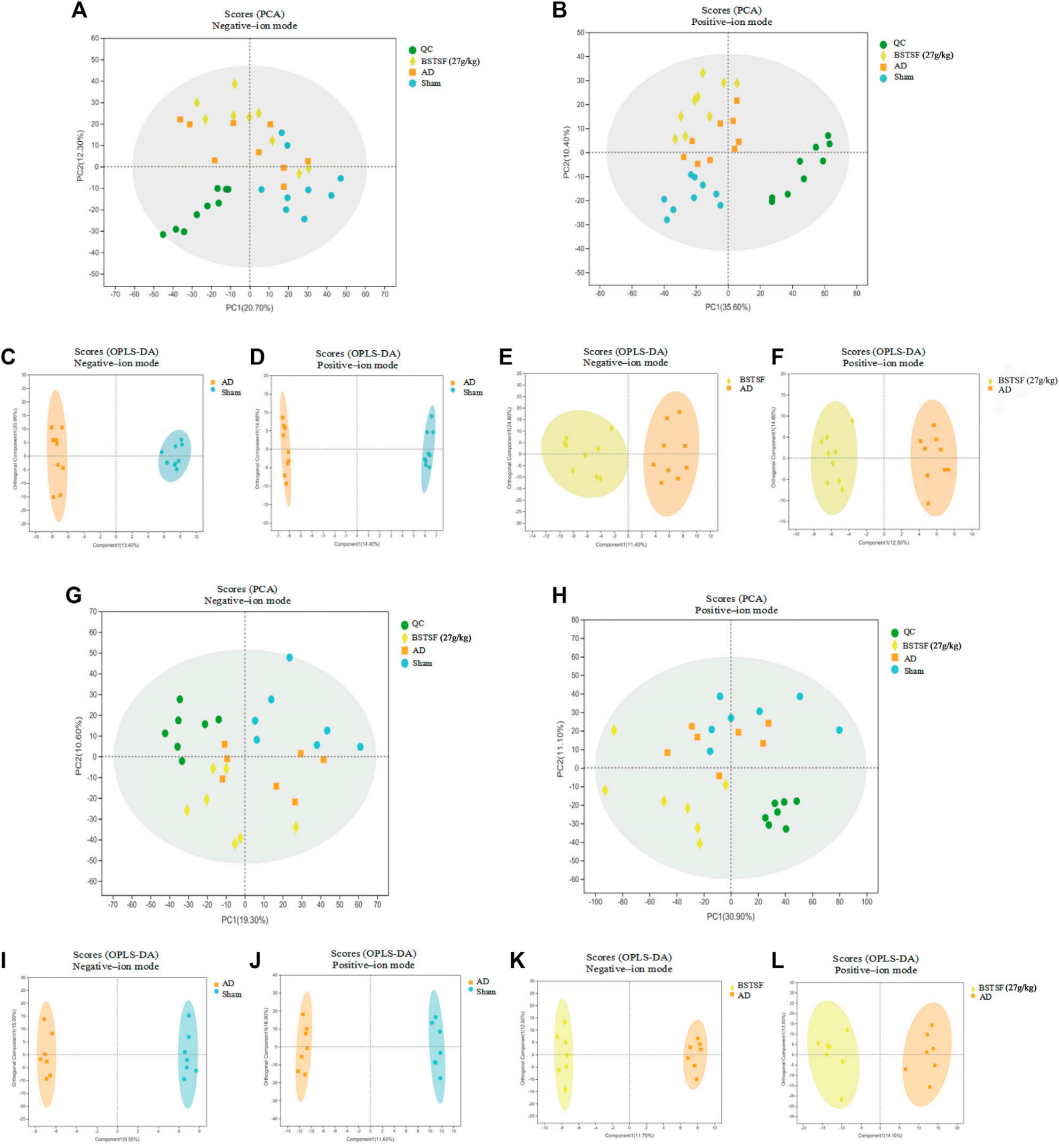
Multivariate data analysis of the cerebral cortex **(A–F)** and hippocampus **(G–L)**. A-B and G–H: PCA score plots in negative-ion mode **(A,G)** and positive-ion mode **(B,H)**; **(C–F)** and **(I–L)**: OPLS-DA score plots between each two groups in positive- and negative-ion modes, respectively.

Based on the pharmacodynamic results, the metabolic profiles of the cerebral cortex and hippocampus in the three groups of rats were analyzed by UPLC-QTOF/MS in both ESI+ and ESI− modes. Then, we performed unsupervised principal component analysis (PCA) and orthogonal partial least square discriminate analysis (OPLS-DA) to evaluate the clustering trend. As shown in the PCA mode, a clear separation was observed between the three groups in both ESI+ and ESI− modes. The QC samples clustered together, thus indicating the stability and reproducibility of the instrumental analysis (Figures 3A,B,G,H). Then, we performed OPLS-DA to refine the separation between the three groups. As shown in an OPLS-DA score plot, there was a clear demarcation between each two groups in ESI + mode and ESI- mode (Figures 3C–F, I–L). The permutation test presented the excellent predictive ability and the models were not overfitting (Supplementary Figure S1). Cross-validated analysis indicated good fitness and predictability without overfitting. These results proved that the AD rat model had successfully been established and that the AD rats had developed metabolic disorders.

#### 3.3.2 Identification of potential biomarkers

First, differentiated metabolites were identified by a variable importance projection (VIP) value > 1.0 and a *p* value <0.05. Approximately 137 and 56 metabolites in the cerebral cortex differed significantly between the AD and sham groups in the ESI+ and ESI− modes, respectively (Supplementary Table S1) while 105 and 40 metabolites in the cerebral cortex differed significantly between the BSTSF and AD groups in the ESI+ and ESI− modes, respectively (Supplementary Table S2). Similarly, in the hippocampus, a total of 134 and 39 differential metabolites were identified between the AD and sham groups in positive and negative ion mode, respectively (Supplementary Table S3). There were 111 and 73 differential metabolites identified between the BSTSF and AD groups in positive and negative ion mode, respectively (Supplementary Table S4).

**FIGURE 4:**
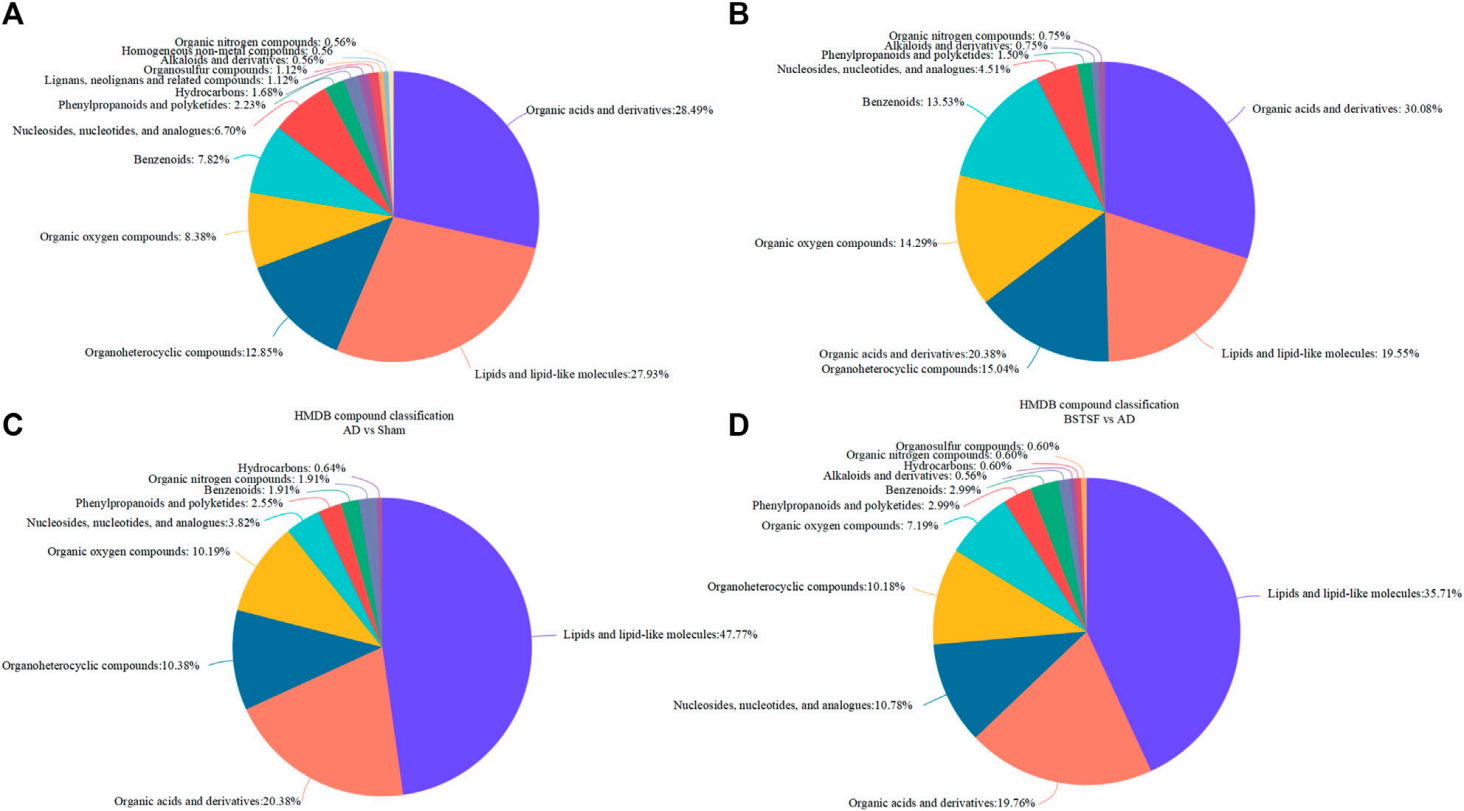
HMDB database classification. **(A,C)**: differential metabolites in the HMDB database classification between the AD and sham groups; **(B,D)**: differential metabolites in the HMDB database classification between the BSTSF and AD groups.

**FIGURE 5:**
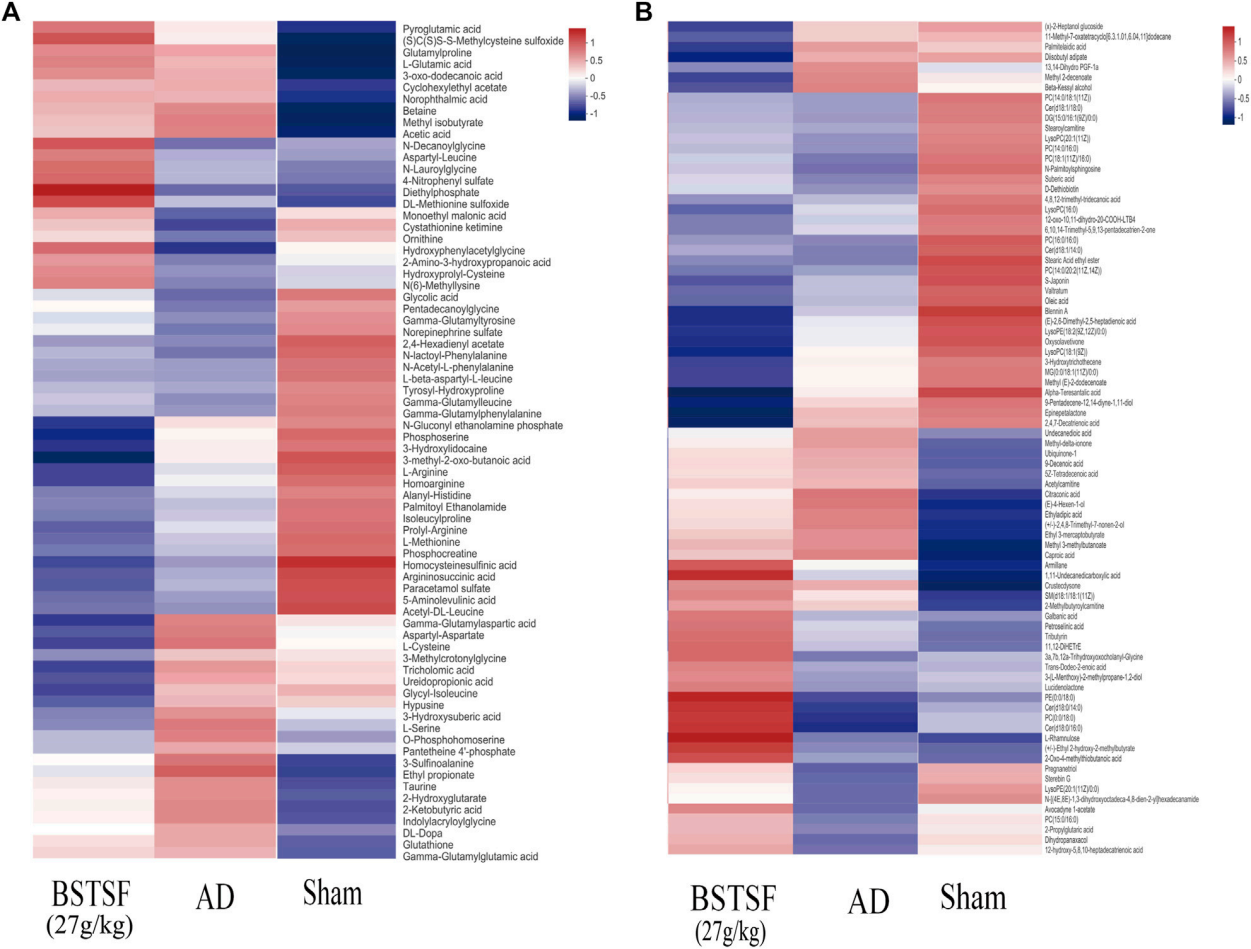
Heat map of differential metabolites in the cerebral cortex. **(A)** organic acid metabolites; **(B)** lipid metabolites.

**FIGURE 6:**
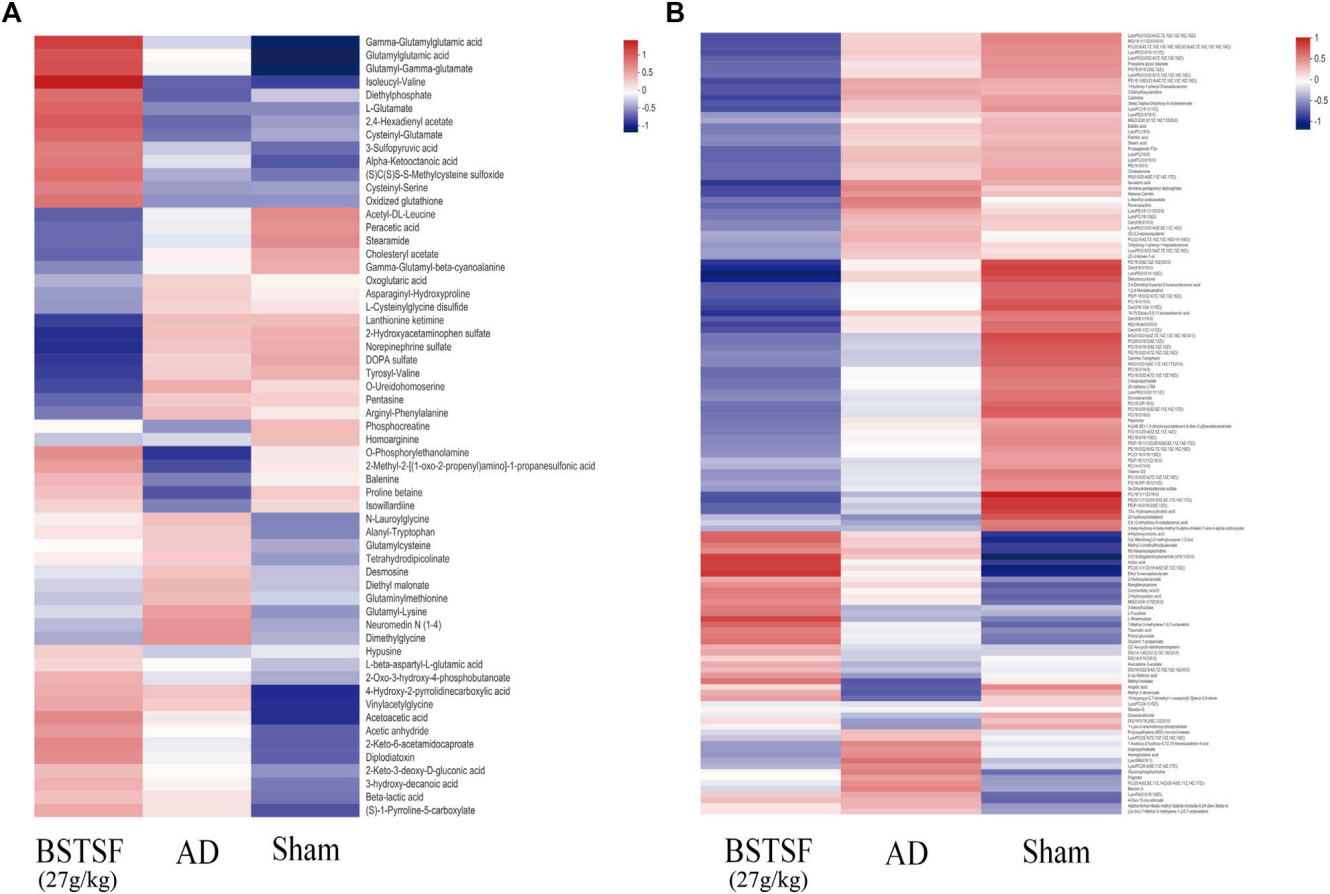
Heat map of differential metabolites in the hippocampus. **(A)** organic acid metabolites; **(B)** lipid metabolites.

Furthermore, the metabolites were classified by the HMDB database. The different colors represent different HMDB classifications while the area represents the relative proportions of metabolites in the classification. As shown in Figure 4A, 28.49% and 27.93% of the differential metabolites between the sham and AD groups were classified as organic acids and lipid metabolites in the cerebral cortex. After BSTSF treatment, 30.08% and 19.55% of the differential metabolites were organic acids and lipids (Figure 4B). In the hippocampus, 20.38% and 47.77% of the differential endogenous metabolites between the AD and sham groups were classified as organic acids and lipid metabolites. 19.76% and 35.71% of the differential metabolites between the BSTSF and AD groups were organic acids and lipids (Figures 4C,D). The classified organic acids and lipid metabolites are presented as a heat map in Figures 5, 6 and Supplementary Table S5,S6.

#### 3.3.3 Metabolic pathway analysis

**FIGURE 7:**
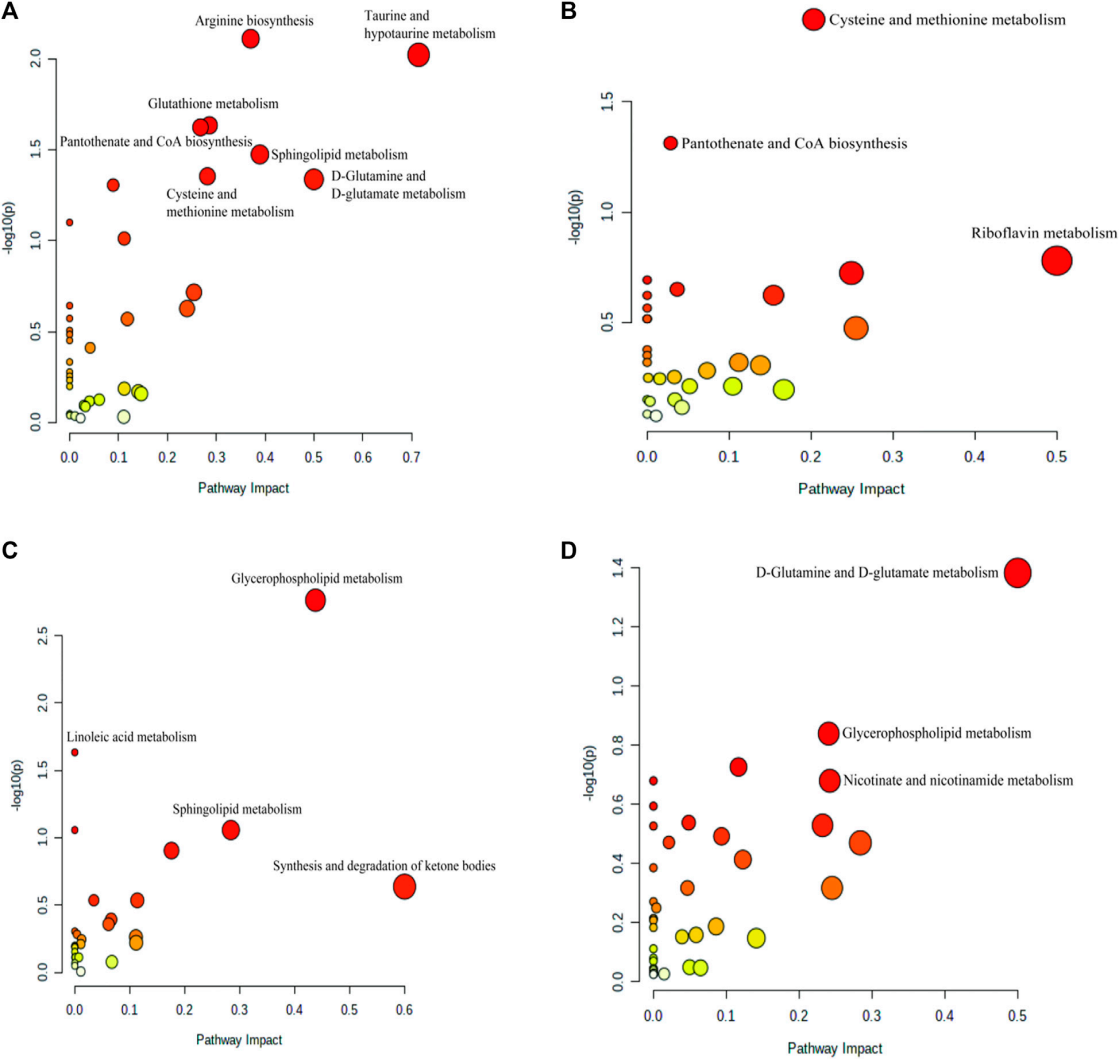
Enriched pathway analysis of the differential metabolites identified in the cerebral cortex **(A,B)** and the hippocampus **(C,D)**. **(A,C)** enriched metabolic pathways between the AD and sham groups; **(B,D)** the enriched metabolic pathways between the BSTSF and AD groups.

To investigate the possible mechanisms underlying the therapeutic effect of BSTSF on AD, we imported the differential metabolites into MetaboAnalyst 5.0 to perform pathway enrichment analysis; related pathways were identified by an impact value >0.1 and *p* <0.05. As shown in Supplementary Table S1, the 193 differential metabolites in the cerebral cortex identified between the sham and AD groups were predominantly involved in arginine biosynthesis, taurine and hypotaurine metabolism, glutathione metabolism, pantothenate and CoA biosynthesis, sphingolipid metabolism, cysteine and methionine metabolism, d-glutamine and d-glutamate metabolism (Supplementary Table S7, Figure 7A). The 145 differential metabolites identified in the cerebral cortex between the BSTSF and AD groups were predominantly involved in cysteine and methionine metabolism (Supplementary Table S7, Figure 7B). In the hippocampus, the differential metabolites between the AD and sham groups were mainly involved in glycerophospholipid metabolism (*p* < 0.05, impact >0.1). The differential metabolites identified between the BSTSF and AD groups were predominantly involved in the d-glutamine and d-glutamate metabolism pathways (*p* < 0.05, impact >0.1) (Supplementary Table S7, Figures 7C,D).

#### 3.3.4 Diagnostic performance of metabolites

**FIGURE 8:**
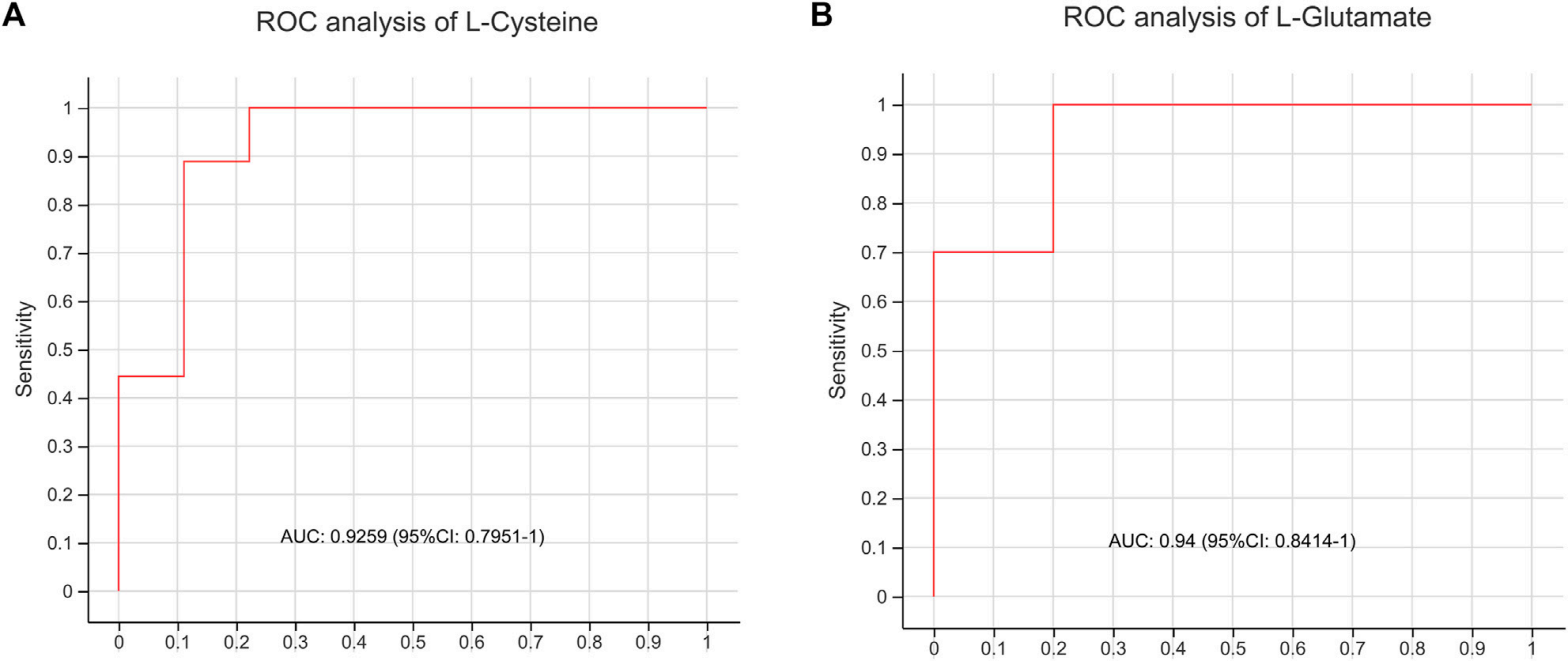
ROC curve analysis of AD group and BSTSF group.

To evaluate whether the differential metabolites could be used to distinguish AD model rats from BSTSF-treated rats, the key metabolites of cysteine and methionine metabolic pathway, as well as glutamine and glutamate metabolic pathway were chosen for classical univariate ROC curve analysis. Our results indicated that L-cysteine and d-glutamine might be used to distinguish AD group from BSTSF group (AUC = 0.9259, 95% CI 0.7951–1, Figure 8A; AUC = 0.94, 95% CI 0.8414–1, Figure 8B).

## 4 Discussion

**FIGURE 9:**
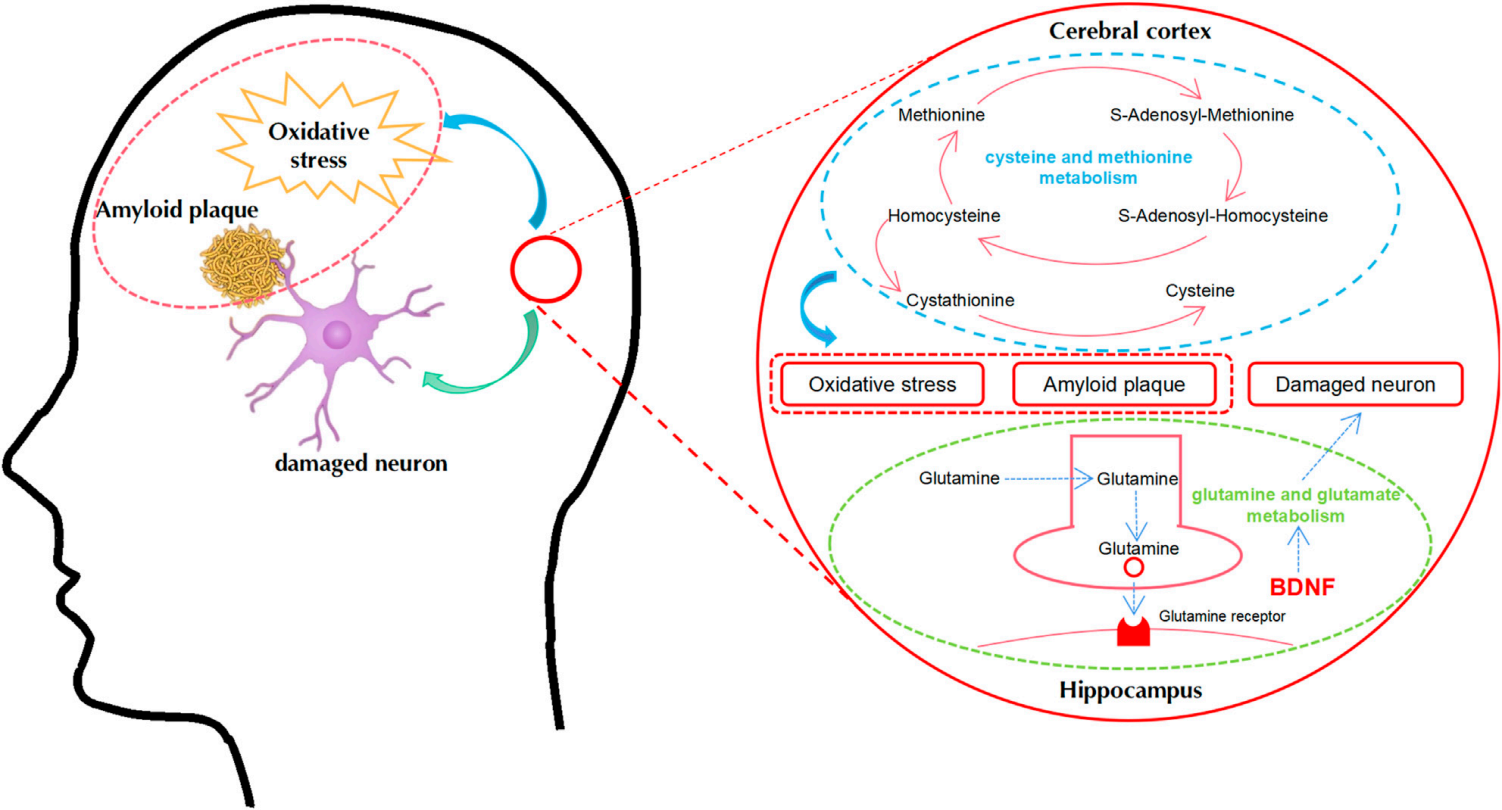
A proposed metabolic pathway explaining the relationship between AD and BSTSF treatment in the cerebral cortex and hippocampus. Blue represents the related metabolic pathways in the cerebral cortex. Green represents the related metabolic pathways in the hippocampus. Abbreviations: BDNF, brain-derived neurotrophic factor.

This represents the first study to comprehensively investigate the mechanisms responsible for the therapeutic action of BSTSF on AD by the integrated metabolomics analysis of the cerebral cortex and hippocampus. Our results showed that Aβ_25-35_-induced AD model rats exhibited cognitive dysfunction and characteristic histopathological changes. Furthermore, there was a distinct metabolomic profile in the cerebral cortex and hippocampus of AD model rats. BSTSF treatment was shown to ameliorate AD-associated cognitive defects and histopathological changes by regulating cysteine and methionine metabolism in the cerebral cortex, along with glutamine and glutamate metabolism in the hippocampus (Figure 9).

A series of previous studies reported alterations in the levels of metabolites in the brains of both AD patients and model mice (Griffin and Bradshaw, 2017; Glenn et al., 2019; Cheng et al., 2021). In this study, a total of 193 metabolites were identified as differential endogenous metabolites with VIP >1 in OPLS-DA and *p* < 0.05 in the cerebral cortex of AD rats. After BSTSF treatment, 145 metabolites were shown to be significantly altered in the AD rats. Furthermore, cysteine and methionine metabolic pathways were identified. It has previously been demonstrated that cysteine and methionine metabolism is associated with neurodegenerative diseases (Abu Ahmad et al., 2019). In our study, the essential metabolic intermediates of cysteine and methionine metabolism showed key alterations; levels of L-methionine were reduced in AD rats, while the levels of L-cysteine and 3-Sulfino-L-alanine were increased. Research suggests that methionine might improve memory deficits and that severe methionine deficiency might cause dementia (Tapia-Rojas et al., 2015). In a previous case-control study, the levels of alanine in the cerebrospinal fluid were significantly increased in the AD group (Mochizuki et al., 1996). Cysteine has also been shown to be upregulated in post-mortem brain frontal cortex samples from AD subjects (Kalecký et al., 2022). After BSTSF intervention, we found that the levels of DL-methionine sulfoxide were increased while those L-cysteine were decreased. Accumulating evidence shows that the AD brain is under extensive oxidative stress (Park et al., 2021; Plascencia-Villa and Perry, 2021; Tamagno et al., 2021) and that methionine is recognized as an important antioxidant (Moskovitz et al., 2016). It has been reported that the formation of Aβ oligomers is attenuated by methionine oxidization and that the methionine oxidized form of Aβ has low toxicity (Fanni et al., 2022). Furthermore, L-cysteine can reduce the self-aggregation of Aβ (Jiang et al., 2020). Our results concurred with these previous findings and consolidated the function of cysteine and methionine metabolism in AD. These results indicated that changes in the levels of cysteine and methionine were rectified after AD rats were treated with BSTSF.

The metabolic pathway regulated by BSTSF in the hippocampus is that responsible for glutamine and glutamate metabolism. Glutamate and glutamine are the most abundant amino acids present in the brain and serve as major energy sources (Cooper and Jeitner, 2016). Glutamate is the main excitatory neurotransmitter in the central nervous system and can influence cognitive function by regulating neuronal growth and synaptic transmission and plasticity (Revett et al., 2013; Andersen et al., 2021). Changes in glutamate metabolism could have a major effect on neural function (Fontana, 2015). In this study, we discovered that the metabolism of d-glutamine and d-glutamate showed differences in the hippocampus when compared between the BSTSF group and AD group. Previous clinical trials and animal research have shown that the levels of glutamine and glutamate were decreased in the brains of both AD patients and animals. For example, a previous study reported that levels of L-glutamate were lower than normal in the cortex of patients who had died with senile dementia of the Alzheimer’s type (Greenamyre et al., 1985). Clinical trials reported that the levels of glutamine and glutamate were significantly reduced in the bilateral hippocampi of patients with AD (Shiino et al., 2012). Furthermore, Lin et al. measured the levels of glutamate in the hippocampus of APP/PS1 mice by using magnetic resonance spectroscopy and discovered that the levels of glutamate were lower in the APP/PS1 group than those in the control group. Furthermore, a recent paper indicated that the availability of L-glutamine decreased in the hippocampus after the chronic administration of scopolamine in animals with induced memory dysfunction (Cieślik et al., 2021). However, electroacupuncture has been shown to increase the levels of glutamate and improve the results of behavioral tests in 12 month-old APP/PS1 mice (Lin et al., 2018). Therefore, it is vital to maintain the glutamate homeostasis in cells to maintain energy metabolism in the brain; dysfunctional energy metabolism has been implicated in many neurodegenerative diseases. In this study, we found that L-glutamate was upregulated in the hippocampus of the BSTSF group. This indicates that BSTSF could ameliorate AD pathology by regulating glutamine and glutamate metabolism. It has been reported that BDNF could regulate the levels of glutamate. Accumulating evidence also demonstrates that BDNF exhibits a protective role against AD pathogenesis (Colucci-D'Amato et al., 2020; de Pins et al., 2019; Liao et al., 2021). BDNF has also been shown to enhance the release of neurotransmitters, including glutamate, *via* various different pathways (Jovanovic et al., 2000; Leal et al., 2014; Numakawa et al., 2014). In our previous study, we found that the levels of BDNF were decreased in the hippocampus of AD rats but increased following treatment with BSTSF (Sheng et al., 2020). Thus, the pathways related to glutamine and glutamate metabolism might play important roles in AD. Further research is now necessary to determine the precise functions and mechanisms of glutamine and glutamate metabolism in the pathological process of AD.

Owing to restrictions associated with the MetaboAnalyst pathway analysis, several important metabolites that are known to be highly related to AD were not included in the final pathway. However, we discovered that there was a disturbance of both sphingolipid and glycerophospholipid metabolism in the cerebral cortex and hippocampus of AD rats. Defects in sphingolipid metabolism and glycerophospholipid metabolism have been linked to numerous neurological diseases including AD, Parkinson’s disease and multiple sclerosis (Alaamery et al., 2021). Sphingolipid metabolism and glycerophospholipid metabolism are both forms of phospholipid metabolism. In our previous study, we found changes in the serum levels of phospholipids in AD rats (Zhang et al., 2020). Several previous studies have shown that ceramides, phosphatidylcholine (PC), and phosphatidylethanolamine (PE) levels were increased in AD (Haughey et al., 2010; Filippov et al., 2012; Calzada et al., 2016; Patrick, 2019). Interestingly, the levels of ceramides, PE and PC in the hippocampus were found to be decreased following BSTSF treatment in the present study. This was consistent with our previous studies and emphasized the importance of phospholipid metabolism in AD (Yi et al., 2020). Apolipoprotein E4 is a major determinant of brain phospholipid homeostasis. It is accepted that patients who possess the APOE4 allele, which plays role in amyloid beta aggregation and clearance, have an increased risk for developing AD (Durmaz et al., 2019; Pontifex et al., 2021). Researchers have also discovered that ApoE4 can regulate the expression of BDNF (Sen et al., 2015). Therefore, BSTSF might regulate phospholipid metabolism and BDNF expression *via* ApoE4, to exert therapeutic effects on AD.

Through integrated cerebral cortex and hippocampus metabolic analyses, we discovered that metabolic dysregulation occurred in the cerebral cortex and hippocampus of AD rat and identified the metabolism-related protective effects of BSTSF on AD. The major advantage of untargeted metabolomics is the discovery of novel metabolites in relation to the study context, and is thus considered as hypothesis-generating. Therefore, these potential metabolites in our project, for example L-cysteine and d-glutamine, could give us some hints or directions for further studies aiming at revealing the mechanisms of BSTSF treatment on AD in depth. However, there were some limitations in this study that should be considered. It is evident that there is a small overlap between the metabolites in the cerebral cortex and hippocampus, thus suggesting potential differences in different brain areas in relation to AD pathology. Hence, enhanced metabolomics technologies, such as targeted metabolomics and spatial metabolomics, are now needed to verify these new metabolite biomarkers and their spatial distributions.

## 5 Conclusion

In this study, we investigated the influence of BSTSF treatment on AD by combining metabolic profiling in the cerebral cortex and hippocampus. Many metabolites were shown to be involved in the development of AD and multiple metabolic pathways were significantly associated with AD. BSTSF appears to exert therapeutic effects on AD by regulating cysteine and methionine metabolism, d-glutamine and d-glutamate metabolism, and phospholipid metabolism. Collectively, our findings provide a fundamental basis for the clinical application of BSTSF and provides novel insight for treating AD.

## Data Availability

The original contributions presented in the study are included in the article/Supplementary Material, further inquiries can be directed to the corresponding author.

## Abbreviations

BSTSF, Bushen Tiansui Formula; AD, Alzheimer’s disease; MWM, Morris water maze; TCM, Traditional Chinese Medicine; BDNF, brain-derived neurotrophic factor; LC-MS, liquid chromatography-mass spectrometry; GO, Gene Ontology; KEGG, Kyoto Encyclopedia of Genes and Genomes; QC, quality control; MS, mass spectrometry; OPLS-DA, Orthogonal Partial Least Squares Discrimination Analysis; VIP, variable importance.

## References

[B1] Abu Ahmad N. Raizman M. Weizmann N. Wasek B. Arning E. Bottiglieri T. (2019). Betaine attenuates pathology by stimulating lipid oxidation in liver and regulating phospholipid metabolism in brain of methionine-choline-deficient rats. FASEB J. 33 (8), 9334–9349. 10.1096/fj.201802683R 31120771

[B2] Alaamery M. Albesher N. Aljawini N. Alsuwailm M. Massadeh S. Wheeler M. A. (2021). Role of sphingolipid metabolism in neurodegeneration. J. Neurochem. 158 (1), 25–35. 10.1111/jnc.15044 32402091 PMC7665988

[B3] Andersen J. V. Markussen K. H. Jakobsen E. Schousboe A. Waagepetersen H. S. Rosenberg P. A. (2021). Glutamate metabolism and recycling at the excitatory synapse in health and neurodegeneration. Neuropharmacology 196, 108719. 10.1016/j.neuropharm.2021.108719 34273389

[B4] Belfiore R. Rodin A. Ferreira E. Velazquez R. Branca C. Caccamo A. (2019). Temporal and regional progression of Alzheimer's disease-like pathology in 3xTg-AD mice. Aging Cell 18 (1), e12873. 10.1111/acel.12873 30488653 PMC6351836

[B5] Calzada E. Onguka O. Claypool S. M. (2016). Phosphatidylethanolamine metabolism in health and disease. Int. Rev. Cell Mol. Biol. 321, 29–88. 10.1016/bs.ircmb.2015.10.001 26811286 PMC4778737

[B6] Chen P. Shen Z. Wang Q. Zhang B. Zhuang Z. Lin J. (2021). Reduced cerebral glucose uptake in an alzheimer's rat model with glucose-weighted chemical exchange saturation transfer imaging. Front. Aging Neurosci. 13, 618690. 10.3389/fnagi.2021.618690 33815088 PMC8010663

[B7] Cheng Y.-J. Lin C.-H. Lane H.-Y. (2021). d-Amino Acids and pLG72 in Alzheimer's disease and schizophrenia. Int. J. Mol. Sci. 22 (20), 10917. 10.3390/ijms222010917 34681579 PMC8535920

[B8] Cieślik P. Siekierzycka A. Radulska A. Płoska A. Burnat G. Brański P. (2021). Nitric oxide-dependent mechanisms underlying MK-801- or scopolamine-induced memory dysfunction in animals: mechanistic studies. Int. J. Mol. Sci. 22 (22), 12282. 10.3390/ijms222212282 34830164 PMC8624219

[B9] Colucci-D'Amato L. Speranza L. Volpicelli F. (2020). Neurotrophic Factor BDNF, Physiological functions and therapeutic potential in depression, neurodegeneration and brain cancer. Int. J. Mol. Sci. 21 (20), E7777. 10.3390/ijms21207777 33096634 PMC7589016

[B10] Cooper A. J. L. Jeitner T. M. (2016). Central Role of glutamate metabolism in the maintenance of nitrogen homeostasis in normal and hyperammonemic brain. Biomolecules 6 (2), E16. 10.3390/biom6020016 27023624 PMC4919911

[B11] Crist A. M. Hinkle K. M. Wang X. Moloney C. M. Matchett B. J. Labuzan S. A. (2021). Transcriptomic analysis to identify genes associated with selective hippocampal vulnerability in Alzheimer's disease. Nat. Commun. 12 (1), 2311. 10.1038/s41467-021-22399-3 33875655 PMC8055900

[B12] Cui L. Lu H. Lee Y. H. (2018). Challenges and emergent solutions for LC-MS/MS based untargeted metabolomics in diseases. Mass Spectrom. Rev. 37 (6), 772–792. 10.1002/mas.21562 29486047

[B13] de Pins B. Cifuentes-Díaz C. Farah A. T. López-Molina L. Montalban E. Sancho-Balsells A. (2019). Conditional BDNF delivery from astrocytes rescues memory deficits, spine density, and synaptic properties in the 5xFAD mouse model of Alzheimer disease. J. Neurosci. 39 (13), 2441–2458. 10.1523/JNEUROSCI.2121-18.2019 30700530 PMC6435824

[B14] Dinel A.-L. Lucas C. Guillemet D. Layé S. Pallet V. Joffre C. (2020). Chronic supplementation with a mix of salvia officinalis and salvia lavandulaefolia improves Morris water maze learning in normal adult C57Bl/6J mice. Nutrients 12 (6), E1777. 10.3390/nu12061777 32549250 PMC7353372

[B15] Durmaz A. Kumral E. Durmaz B. Onay H. Aslan G. I. Ozkinay F. (2019). Genetic factors associated with the predisposition to late onset Alzheimer's disease. Gene 707, 212–215. 10.1016/j.gene.2019.05.030 31102717

[B16] Fanni A. M. Okoye D. Monge F. A. Hammond J. Maghsoodi F. Martin T. D. (2022). Controlled and selective photo-oxidation of amyloid-β fibrils by oligomeric-phenylene tthynylenes. ACS Appl. Mat. Interfaces 14, 14871. 10.1021/acsami.1c22869 PMC10452927 35344326

[B17] Filippov V. Song M. A. Zhang K. Vinters H. V. Tung S. Kirsch W. M. (2012). Increased ceramide in brains with Alzheimer's and other neurodegenerative diseases. J. Alzheimers Dis. 29 (3), 537–547. 10.3233/JAD-2011-111202 22258513 PMC3643694

[B18] Fontana A. C. K. (2015). Current approaches to enhance glutamate transporter function and expression. J. Neurochem. 134 (6), 982–1007. 10.1111/jnc.13200 26096891

[B19] Glenn J. M. Madero E. N. Bott N. T. (2019). Dietary protein and amino acid intake: links to the maintenance of cognitive health. Nutrients 11 (6), E1315. 10.3390/nu11061315 31212755 PMC6627761

[B20] Greenamyre J. T. Penney J. B. Young A. B. D'Amato C. J. Hicks S. P. Shoulson I. (1985). Alterations in L-glutamate binding in Alzheimer's and Huntington's diseases. Science 227 (4693), 1496–1499. 10.1126/science.2858129 2858129

[B21] Griffin J. W. D. Bradshaw P. C. (2017). Amino acid catabolism in alzheimer's disease brain: friend or foe? Oxid. Med. Cell. Longev. 2017, 5472792. 10.1155/2017/5472792 28261376 PMC5316456

[B22] Haughey N. J. Bandaru V. V. R. Bae M. Mattson M. P. (2010). Roles for dysfunctional sphingolipid metabolism in Alzheimer's disease neuropathogenesis. Biochim. Biophys. Acta 1801 (8), 878–886. 10.1016/j.bbalip.2010.05.003 20452460 PMC2907186

[B23] Hou Z. Li F. Chen J. Liu Y. He C. Wang M. (2019). Beneficial effects of sagacious Confucius' Pillow Elixir on cognitive function in senescence-accelerated P8 mice (SAMP8) via the NLRP3/caspase-1 pathway. Evid. Based. Complement. Altern. Med. 2019, 3097923. 10.1155/2019/3097923 PMC6874996 31781266

[B24] Hui S. Yang Y. Peng W.-J. Sheng C.-X. Gong W. Chen S. (2017). Protective effects of Bushen Tiansui decoction on hippocampal synapses in a rat model of Alzheimer's disease. Neural Regen. Res. 12 (10), 1680–1686. 10.4103/1673-5374.217347 29171433 PMC5696849

[B25] Jiang W. YanYuYao D. Fei X. Ai L. Di Y. Zhang J. (2020). Single point mutation from E22-to-K in A initiates early-onset alzheimer's disease by binding with catalase. Oxid. Med. Cell Longev. 2020, 4981204. 10.1155/2020/4981204 33425208 PMC7775154

[B26] Jovanovic J. N. Czernik A. J. Fienberg A. A. Greengard P. Sihra T. S. (2000). Synapsins as mediators of BDNF-enhanced neurotransmitter release. Nat. Neurosci. 3 (4), 323–329. 10.1038/73888 10725920

[B27] Kalecký K. Ashcraft P. Bottiglieri T. (2022). One-carbon metabolism in alzheimer's disease and Parkinson's disease brain tissue. Nutrients 14 (3), 599. 10.3390/nu14030599 35276958 PMC8838558

[B28] Lazarov O. Hollands C. (2016). Hippocampal neurogenesis: Learning to remember. Prog. Neurobiol. 138-140, 1–18. 10.1016/j.pneurobio.2015.12.006 26855369 PMC4828289

[B29] Leal G. Comprido D. Duarte C. B. (2014). BDNF-induced local protein synthesis and synaptic plasticity. Neuropharmacology 76 Pt C, 639–656. 10.1016/j.neuropharm.2013.04.005 23602987

[B30] Liao J. Chen C. Ahn E. H. Liu X. Li H. Edgington-Mitchell L. E. (2021). Targeting both BDNF/TrkB pathway and delta-secretase for treating Alzheimer's disease. Neuropharmacology 197, 108737. 10.1016/j.neuropharm.2021.108737 34343610 PMC8478860

[B31] Lin R. Li L. Zhang Y. Huang S. Chen S. Shi J. (2018). Electroacupuncture ameliorate learning and memory by improving N-acetylaspartate and glutamate metabolism in APP/PS1 mice. Biol. Res. 51 (1), 21. 10.1186/s40659-018-0166-7 29980225 PMC6034239

[B32] Liu X. Wei F. Liu H. Zhao S. Du G. Qin X. (2021). Integrating hippocampal metabolomics and network pharmacology deciphers the antidepressant mechanisms of Xiaoyaosan. J. Ethnopharmacol. 268, 113549. 10.1016/j.jep.2020.113549 33152435

[B33] Mochizuki Y. Oishi M. Hara M. Takasu T. (1996). Amino acid concentration in dementia of the Alzheimer type and multi-infarct dementia. Ann. Clin. Lab. Sci. 26 (3), 275–278. 8726221

[B34] Moskovitz J. Du F. Bowman C. F. Yan S. S. (2016). Methionine sulfoxide reductase A affects β-amyloid solubility and mitochondrial function in a mouse model of Alzheimer's disease. Am. J. Physiol. Endocrinol. Metab. 310 (6), E388–E393. 10.1152/ajpendo.00453.2015 26786779 PMC4796266

[B35] Numakawa T. Matsumoto T. Ooshima Y. Chiba S. Furuta M. Izumi A. (2014). Impairments in brain-derived neurotrophic factor-induced glutamate release in cultured cortical neurons derived from rats with intrauterine growth retardation: possible involvement of suppression of TrkB/phospholipase C-γ activation. Neurochem. Res. 39 (4), 785–792. 10.1007/s11064-014-1270-x 24599793

[B36] Park M. W. Cha H. W. Kim J. Kim J. H. Yang H. Yoon S. (2021). NOX4 promotes ferroptosis of astrocytes by oxidative stress-induced lipid peroxidation via the impairment of mitochondrial metabolism in Alzheimer's diseases. Redox Biol. 41, 101947. 10.1016/j.redox.2021.101947 33774476 PMC8027773

[B37] Patrick R. P. (2019). Role of phosphatidylcholine-DHA in preventing APOE4-associated Alzheimer's disease. FASEB J. 33 (2), 1554–1564. 10.1096/fj.201801412R 30289748 PMC6338661

[B38] Plascencia-Villa G. Perry G. (2021). Preventive and therapeutic strategies in alzheimer's disease: focus on oxidative stress, redox metals, and ferroptosis. Antioxid. Redox Signal. 34 (8), 591–610. 10.1089/ars.2020.8134 32486897 PMC8098758

[B39] Pontifex M. G. Martinsen A. Saleh R. N. M. Harden G. Tejera N. Müller M. (2021). APOE4 genotype exacerbates the impact of menopause on cognition and synaptic plasticity in APOE-TR mice. FASEB J. 35 (5), e21583. 10.1096/fj.202002621RR 33891334

[B40] Revett T. J. Baker G. B. Jhamandas J. Kar S. (2013). Glutamate system, amyloid ß peptides and tau protein: functional interrelationships and relevance to alzheimer disease pathology. J. Psychiatry Neurosci. 38 (1), 6–23. 10.1503/jpn.110190 22894822 PMC3529221

[B41] Sen A. Nelson T. J. Alkon D. L. (2015). ApoE4 and Aβ oligomers reduce BDNF expression via HDAC nuclear translocation. J. Neurosci. 35 (19), 7538–7551. 10.1523/JNEUROSCI.0260-15.2015 25972179 PMC6705431

[B42] Serneels L. T'Syen D. Perez-Benito L. Theys T. Holt M. G. De Strooper B. (2020). Modeling the β-secretase cleavage site and humanizing amyloid-beta precursor protein in rat and mouse to study Alzheimer's disease. Mol. Neurodegener. 15 (1), 60. 10.1186/s13024-020-00399-z 33076948 PMC7574558

[B43] Shao J. Liu Y. Wang H. Luo Y. Chen L. (2020). An integrated fecal microbiome and metabolomics in T2DM rats reveal antidiabetes effects from host-microbial metabolic Axis of EtOAc extract from Sophora flavescens. Oxid. Med. Cell. Longev. 2020, 1805418. 10.1155/2020/1805418 32566075 PMC7273480

[B44] Sheng C. Xu P. Liu X. Peng W. Xiang D. Luo S. (2020). Bushen-tiansui formula improves cognitive functions in an Aβ 1-42 fibril-infused rat model of alzheimer's disease. Neural Plast. 2020, 8874885. 10.1155/2020/8874885 33029123 PMC7532368

[B45] Shi W. Yuan X. Cui K. Li H. Fu P. Rehman S. U. (2021). LC-MS/MS based metabolomics reveal candidate biomarkers and metabolic changes in different buffalo species. Animals. 11 (2), 560. 10.3390/ani11020560 33672725 PMC7924386

[B46] Shiino A. Watanabe T. Shirakashi Y. Kotani E. Yoshimura M. Morikawa S. (2012). The profile of hippocampal metabolites differs between Alzheimer's disease and subcortical ischemic vascular dementia, as measured by proton magnetic resonance spectroscopy. J. Cereb. Blood Flow. Metab. 32 (5), 805–815. 10.1038/jcbfm.2012.9 22314267 PMC3345919

[B47] Su L. Li R. Zhang Z. Liu J. Du J. Wei H. (2022). Identification of altered exosomal microRNAs and mRNAs in Alzheimer's disease. Ageing Res. Rev. 73, 101497. 10.1016/j.arr.2021.101497 34710587

[B48] Tamagno E. Guglielmotto M. Vasciaveo V. Tabaton M. (2021). Oxidative stress and beta amyloid in alzheimer's disease. Which comes first: the chicken or the egg? Antioxidants (Basel) 10 (9), 1479. 10.3390/antiox10091479 34573112 PMC8468973

[B49] Tapia-Rojas C. Lindsay C. B. Montecinos-Oliva C. Arrazola M. S. Retamales R. M. Bunout D. (2015). Is L-methionine a trigger factor for Alzheimer's-like neurodegeneration?: Changes in Aβ oligomers, tau phosphorylation, synaptic proteins, Wnt signaling and behavioral impairment in wild-type mice. Mol. Neurodegener. 10, 62. 10.1186/s13024-015-0057-0 26590557 PMC4654847

[B50] Xia Z. Peng W. Cheng S. Zhong B. Sheng C. Zhang C. (2017). Naoling decoction restores cognitive function by inhibiting the neuroinflammatory network in a rat model of Alzheimer's disease. Oncotarget 8 (26), 42648–42663. 10.18632/oncotarget.17337 28487495 PMC5522095

[B51] Yi M. Zhang C. Zhang Z. Yi P. Xu P. Huang J. (2020). Integrated metabolomic and lipidomic analysis reveals the neuroprotective mechanisms of Bushen Tiansui formula in an A1-42-induced rat model of Alzheimer's disease. Oxid. Med. Cell. Longev. 2020, 5243453. 10.1155/2020/5243453 32655770 PMC7322593

[B52] Zhang Z. Yi P. Yang J. Huang J. Xu P. Hu M. (2020). Integrated network pharmacology analysis and serum metabolomics to reveal the cognitive improvement effect of Bushen Tiansui formula on Alzheimer's disease. J. Ethnopharmacol. 249, 112371. 10.1016/j.jep.2019.112371 31683034

[B53] Zheng Y. Liu A. Wang Z.-J. Cao Q. Wang W. Lin L. (2019). Inhibition of EHMT1/2 rescues synaptic and cognitive functions for Alzheimer's disease. Brain 142 (3), 787–807. 10.1093/brain/awy354 30668640 PMC6391616

