# Supplementary material for: Untargeted metabolomics analysis of the hippocampus and cerebral cortex identified the neuroprotective mechanisms of Bushen Tiansui formula in an aβ25-35-induced rat model of Alzheimer’s disease
Source: Front Pharmacol. 2022 Oct 20;13:990307. doi: 10.3389/fphar.2022.990307 (PMC9630565; doi:10.3389/fphar.2022.990307)
Supplement: Supplementary file 8 [file DataSheet1.docx]

1. Metabolite pathway changes between AD and Sham groups in the cerebral cortex.

| No. | Pathway | Total | Hits | Raw *p* | −Log(*p*) | FDR *p* | Impact |
| --- | --- | --- | --- | --- | --- | --- | --- |
| 1 | Arginine biosynthesis | 14 | 4 | 0.0078 | 2.1096 | 0.4000 | 0.3706 |
| 2 | Taurine and hypotaurine metabolism | 8 | 3 | 0.0095 | 2.0211 | 0.4000 | 0.7143 |
| 3 | Glutathione metabolism | 28 | 5 | 0.0232 | 1.6336 | 0.5009 | 0.2861 |
| 4 | Pantothenate and CoA biosynthesis | 19 | 4 | 0.0239 | 1.6224 | 0.5009 | 0.2679 |
| 5 | Sphingolipid metabolism | 21 | 4 | 0.0336 | 1.4738 | 0.5200 | 0.3895 |
| 6 | Cysteine and methionine metabolism | 33 | 5 | 0.0443 | 1.3538 | 0.5200 | 0.2821 |
| 7 | D-Glutamine and D-glutamate metabolism | 6 | 2 | 0.0460 | 1.3370 | 0.5200 | 0.5000 |
| 8 | Glycine, serine and threonine metabolism | 34 | 5 | 0.0495 | 1.3052 | 0.5200 | 0.0891 |
| 9 | Valine, leucine and isoleucine biosynthesis | 8 | 2 | 0.0794 | 1.1001 | 0.7413 | 0.0000 |
| 10 | Purine metabolism | 66 | 7 | 0.0973 | 1.0117 | 0.8177 | 0.1118 |

2. Metabolite pathway changes between BSTSF and AD groups in the cerebral cortex.

| No. | Pathway | Total | Hits | Raw p | −Log(*p*) | FDR p | Impact |
| --- | --- | --- | --- | --- | --- | --- | --- |
| 1 | Cysteine and methionine metabolism | 33 | 5 | 0.0134 | 1.8731 | 1.0000 | 0.2032 |
| 2 | Pantothenate and CoA biosynthesis | 19 | 3 | 0.0487 | 1.3124 | 1.0000 | 0.0286 |
| 3 | Riboflavin metabolism | 4 | 1 | 0.1663 | 0.7792 | 1.0000 | 0.5000 |
| 4 | Glycine, serine and threonine metabolism | 34 | 3 | 0.1891 | 0.7233 | 1.0000 | 0.2492 |
| 5 | Linoleic acid metabolism | 5 | 1 | 0.2034 | 0.6917 | 1.0000 | 0.0000 |
| 6 | Amino sugar and nucleotide sugar metabolism | 37 | 3 | 0.2242 | 0.6493 | 1.0000 | 0.0366 |
| 7 | Sphingolipid metabolism | 21 | 2 | 0.2385 | 0.6226 | 1.0000 | 0.1542 |
| 8 | D-Glutamine and D-glutamate metabolism | 6 | 1 | 0.2389 | 0.6218 | 1.0000 | 0.0000 |
| 9 | Thiamine metabolism | 7 | 1 | 0.2728 | 0.5642 | 1.0000 | 0.0000 |
| 10 | Valine, leucine and isoleucine biosynthesis | 8 | 1 | 0.3052 | 0.5154 | 1.0000 | 0.0000 |

3. Metabolite pathway changes between AD and Sham groups in the hippocampus.

| No. | Pathway | Total | Hits | Raw *p* | −Log(*p*) | FDR *p* | Impact |
| --- | --- | --- | --- | --- | --- | --- | --- |
| 1 | Glycerophospholipid metabolism | 36 | 7 | 0.0017 | 2.7629 | 0.1450 | 0.4379 |
| 2 | Linoleic acid metabolism | 5 | 2 | 0.0233 | 1.6336 | 0.9765 | 0.0000 |
| 3 | beta-Alanine metabolism | 21 | 3 | 0.0877 | 1.0570 | 1.0000 | 0.0000 |
| 4 | Sphingolipid metabolism | 21 | 3 | 0.0877 | 1.0570 | 1.0000 | 0.2840 |
| 5 | Arginine and proline metabolism | 38 | 4 | 0.1246 | 0.9045 | 1.0000 | 0.1759 |
| 6 | Synthesis and degradation of ketone bodies | 5 | 1 | 0.2307 | 0.6370 | 1.0000 | 0.6000 |
| 7 | Pentose phosphate pathway | 21 | 2 | 0.2912 | 0.5358 | 1.0000 | 0.0346 |
| 8 | Amino sugar and nucleotide sugar metabolism | 37 | 3 | 0.2916 | 0.5352 | 1.0000 | 0.1137 |
| 9 | Galactose metabolism | 27 | 2 | 0.4049 | 0.3926 | 1.0000 | 0.0662 |
| 10 | Purine metabolism | 66 | 4 | 0.4383 | 0.3583 | 1.0000 | 0.0614 |

4. Metabolite pathway changes between BSTSF and AD groups in the hippocampus.

| No. | Pathway | Total | Hits | Raw *p* | −Log(*p*) | FDR *p* | Impact |
| --- | --- | --- | --- | --- | --- | --- | --- |
| 1 | D-Glutamine and D-glutamate metabolism | 6 | 2 | 0.0415 | 1.3824 | 1.0000 | 0.5000 |
| 2 | Glycerophospholipid metabolism | 36 | 4 | 0.1448 | 0.8393 | 1.0000 | 0.2405 |
| 3 | Arginine biosynthesis | 14 | 2 | 0.1877 | 0.7265 | 1.0000 | 0.1168 |
| 4 | Butanoate metabolism | 15 | 2 | 0.2090 | 0.6799 | 1.0000 | 0.0000 |
| 5 | Nicotinate and nicotinamide metabolism | 15 | 2 | 0.2090 | 0.6799 | 1.0000 | 0.2421 |
| 6 | Linoleic acid metabolism | 5 | 1 | 0.2546 | 0.5942 | 1.0000 | 0.0000 |
| 7 | Cysteine and methionine metabolism | 33 | 3 | 0.2895 | 0.5384 | 1.0000 | 0.0484 |
| 8 | Pantothenate and CoA biosynthesis | 19 | 2 | 0.2956 | 0.5294 | 1.0000 | 0.2321 |
| 9 | Nitrogen metabolism | 6 | 1 | 0.2972 | 0.5270 | 1.0000 | 0.0000 |
| 10 | Purine metabolism | 66 | 5 | 0.3219 | 0.4923 | 1.0000 | 0.0935 |

Hits: the matched number of metabolites in one pathway. Raw *p*: the original P value calculated from the enrichment analysis. FDR *p*: the P value adjusted using false discovery rate.
